# The enigma of cell intercalation

**DOI:** 10.7554/eLife.98052

**Published:** 2024-05-03

**Authors:** Raphaël Clément

**Affiliations:** 1 Institut de Biologie du Développement de Marseille, Aix Marseille University, CNRS Marseille France

**Keywords:** development, tissue morphogenesis, cell intercalation, convergent extension, geometry, *D. melanogaster*

## Abstract

Geometric criteria can be used to assess whether cell intercalation is active or passive during the convergent extension of tissue.

**Related research article** Brauns F, Claussen NH, Wieschaus EF, Shraiman BI. 2024. The geometric basis of epithelial convergent extension. *eLife* **13**:RP95521. doi: 10.7554/eLife.95521.

During embryonic development, living tissues undergo a range of transformations, including massive shape changes. This process, known as morphogenesis, can occur through growth, cell division or cell death, but also through the coordination of internal forces generated by individual cells. Thanks to advances in microscopy and physical biology, much is known about how cells produce such forces and how they integrate on a larger scale to drive robust changes in tissue shape (Heisenberg and Bellaïche, 2013). Yet, it remains a challenge to disentangle which part of the deformation is due to internal forces produced within the considered tissue, and which part is due to the movement of adjacent regions.

Now, in eLife, Fridtjof Brauns, Nikolas Claussen, Eric Wieschaus, and Boris Shraiman report new insights into the mechanisms of tissue convergent extension (Brauns et al., 2024), a process whereby tissues elongate along one axis while contracting along the other, resulting in a longer and narrower shape. Convergent extension is associated with intercalation events during which quartets of cells exchange neighbors. In this process, two cells lose contact with one another, while the other two gain contact as the central interface collapses (Figure 1A). Intercalation allows tissue extension to proceed without accumulating strain at the cellular level. Whether intercalation passively follows tissue extension (for instance caused by another adjacent active region), or is itself an active driver of tissue extension, remains difficult to assess.

**Figure 1.**
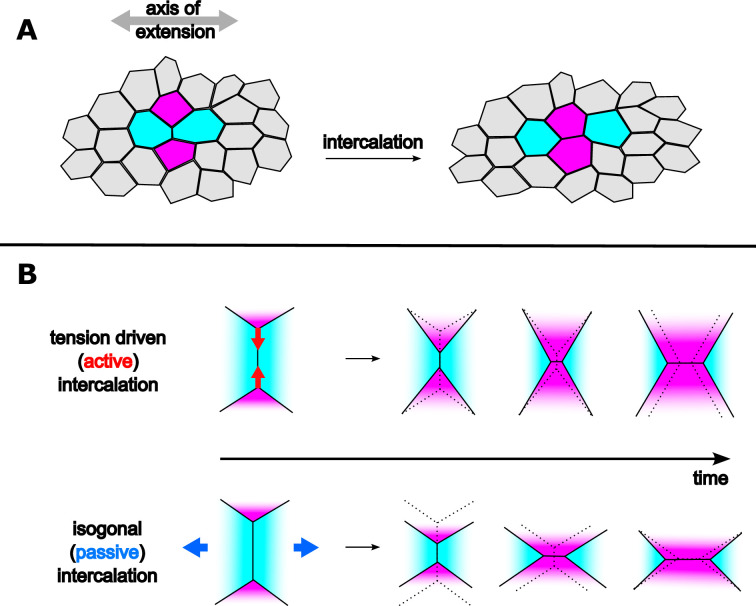
Illustration of cell intercalation during convergent extension of tissue. (**A**) Intercalating quartet of cells in an epithelial tissue. Two cells gain contact (magenta), and two cells lose contact (cyan) as the central interface collapses. (**B**) Tension driven (active) versus isogonal (passive) intercalation.

To investigate, the researchers, based at the University of California Santa Barbara and Princeton University, developed a quantitative, model-based analysis framework to study previously published data on fruit fly embryonic development. They focussed on two regions undergoing intercalation events: the germband (an epithelial monolayer that eventually develops into the segmented trunk of the embryo), and the amnioserosa (an extraembryonic epithelial monolayer). They used a technique, called force inference, on intercalating quartets, which allows inferring the tension at cell interfaces based on cell geometry (Roffay et al., 2021). This revealed that collapsing interfaces located between the two cells about to connect, display radically distinct tension dynamics in the germband and the amnioserosa. In the germband, in which myosin activity (a force-producing protein) is known to actively drive intercalation events (Rauzi et al., 2008), tension increases before the interface collapses. In the amnioserosa, however, tension remains constant until collapse.

To make sense of this contrast, Brauns et al. decomposed the strain of intercalating quartets into two components. The passive, isogonal component allows the quartet to deform, such as getting wider or taller, whilst maintaining the same angles, and reflects external forces exerted by adjacent tissues. The active tension component affects how the edges of the quartet are angled, and reflects variations of local interface tensions. During intercalation in the germband, the isogonal component remains constant, while the tension component increases, confirming that active changes in tension drive intercalation. The opposite is found for the amnioserosa. Active tension and passive isogonal strain thus appear as distinct ways to enable cell neighbor exchange in elongating tissues (Figure 1B), with distinct geometric signatures.

Brauns et al. then propose a minimal model in line with recent studies (Dierkes et al., 2014; Sknepnek et al., 2023), which postulates that the amount of myosin increases as tension grows during the collapse of cell interfaces (in other words, a positive feedback on tension). It further assumes that passive tension is dissipated by the turnover of cytoskeletal crosslinkers, which agrees with previous observations (Clément et al., 2017; Khalilgharibi et al., 2019). The absence of the positive tension feedback corresponds to the case of passive intercalation under external stretch. The model qualitatively recapitulates their findings at the quartet scale.

Finally, Brauns et al. compute tension anisotropy and isogonal strain at the embryo scale, which allows the regions of the tissue undergoing active or passive intercalation to be identified. They show that ‘tension bridge’ arrangements (an interface with high tension surrounded by lower tension interfaces) are more efficient at driving intercalation than ‘tension cable’ arrangements (adjacent interfaces aligned due to similarly high levels of tension), because tension bridges require less tension anisotropy to achieve an exchange of neighboring cells.

Overall, the elegant work of Brauns et al. proposes simple geometric criteria for assessing whether intercalation is active or passive and makes explicit the conditions for intercalation. However, important questions remain. First, the tissue-scale analysis remains essentially qualitative at this stage. Second, it has been reported that germband extension can still proceed when the rate of intercalation is significantly reduced, or even in the absence of myosin polarity. Conversely, mutant embryos that fail to achieve posterior midgut invagination, which was shown to pull on the germband, also fail to extend the germband, even though myosin polarity is preserved (Collinet et al., 2015). This suggests that intercalation – even active – might support extension rather than drive it. It seems that convergent extension, the alpha and omega of tissue morphogenesis, still holds mysteries for the years to come.
